# The relevance of organelle interactions in cellular senescence

**DOI:** 10.7150/thno.70588

**Published:** 2022-02-28

**Authors:** Jiewu Huang, Ping Meng, Cheng Wang, Yunfang Zhang, Lili Zhou

**Affiliations:** 1 State Key Laboratory of Organ Failure Research, National Clinical Research Center for Kidney Disease, Guangdong Provincial Clinical Research Center for Kidney Disease, Guangdong Provincial Key Laboratory of Nephrology, Division of Nephrology, Nanfang Hospital, Southern Medical University, Guangzhou, China.; 2 Department of Central Laboratory, Huadu District People's Hospital, Southern Medical University, Guangzhou, China.; 3 Division of nephrology, Department of medicine, the Fifth affiliated hospital of Sun Yat-Sen University, Zhuhai, Guangdong, China.; 4 Department of Nephrology, Huadu District People's Hospital, Southern Medical University, Guangzhou, China.; 5 Bioland Laboratory (Guangzhou Regenerative Medicine and Health Guangdong Laboratory), Guangzhou, China.

**Keywords:** organelle, interaction, communication, MCSs, cellular senescence

## Abstract

Organelles are tiny structures with specific functions in eukaryotic cells. Since they are covered with membranes, different organelles can perform biological processes that are incompatible. Organelles can also actively communicate with each other to maintain cellular homeostasis* via* the vesicular trafficking pathways and membrane contact sites (MCSs), which allow the exchange of metabolites and other information required for normal cellular physiology. An imbalance in organelle interactions may result in multiple pathological processes. Growing evidence shows that abnormal organelle communication contributes to cellular senescence and is associated with organ aging. However, the key role of organelle interactions in aging has not yet been broadly reviewed and fully investigated. In this review, we summarize the role of organelle interactions in cellular senescence, and highlight their relevance for cellular calcium homeostasis, protein and lipid homeostasis, and mitochondrial quality control. Our review reveals important mechanisms of organelle interactions in cellular senescence and provides important clues for intervention strategies from a new perspective.

## 1. Introduction

Organelles, including the endoplasmic reticulum (ER), mitochondria, and endosomes, possess membranes that allow organelles to perform incompatible biological processes 1-3. However, organelles also communicate and cooperate to accomplish information exchange and specific functions 1, 2. Organelle interactions can be mediated by vesicle transmission and non-vesicular mechanisms 1, 4. In this study, we focus on the latter. Membrane contact sites (MCSs) are formed by the close contact between two organelle membranes 5. They are a major pathway for organelle interactions and act as hubs for signaling interactions 1, 6. In addition to facilitating ion, lipid, and protein exchange, MCSs also play an important role in organelle dynamics 1, 6. For example, lysosomes and ER both play a role in mitochondrial division through the MCSs 1, 3, 6. MCSs are tethered by the parallel protein-protein or protein-lipid interactions, with a typical distance of 10-30 nm between organelles. MCS tethers are enriched with specific proteins or lipids that affect organelle function 5-8. MCSs are often composed of several tethers, but they are dynamically expressed and regulated by modifications 6.

Cellular senescence can be defined as permanent cell cycle arrest. It plays a key role in embryogenesis, host immunity, tissue homeostasis 9, 10, inflammation, and malignancy 11. Cellular senescence independently contributes to aging and age-related diseases, such as Alzheimer's disease and renal fibrosis 9, 10, 12, 13. Organelle interactions are extensively involved in mitochondrial dysfunction and cellular calcium imbalance, which are the underlying mechanisms of cellular senescence. However, the relevance of organelle interactions in cellular senescence has not yet been elucidated. In this review, we discuss the key role of organelle interactions in cellular senescence and highlight their relevance in cellular energy metabolism. We also comment on intervention strategies to retard cellular senescence by modulating organelle interactions.

## 2. Organelle interactions

Organelle interactions occur across multiple organelles, including the ER, mitochondria, Golgi apparatus, peroxisomes, lipid droplets (LDs), endosomes, and lysosomes. As one of the largest organelles in eukaryotic cells, the ER plays a major role in synthesis, folding, modification, structural maturation, and targeted deployment of cellular proteomes 14, 15, and serves as a central hub of lipid metabolism 14. Mitochondria are the master controllers of energy metabolism 16. They possess two layers of plasma membranes 17, and their own circular genome, referred to as mitochondrial DNA (mtDNA) 17. The Golgi apparatus, located adjacent to the nucleus, is a central membrane-bound organelle for trafficking, processing, and sorting of membrane proteins, secretory proteins, and lipids 18. Peroxisomes have variable functions in different cells 19, ranging from fatty acid β-oxidation and hydrogen peroxide degradation to glycerol metabolism and maintenance of cellular integrity 19. LDs store neutral lipids for energy or membrane synthesis and are primarily located in the cytoplasm 20. There are two types of endosomes: early and late. Early endosomes are the cargo originating from the uptake of extracellular material by the plasma membrane (PM), which can be transported into the PM, ER, or late endosomes. Late endosomes go into lysosomes either *via* direct acidification or fusion with existing lysosomes 21. Lysosomes digest extracellular or cell-surface cargos obtained *via* endocytosis or degrade intracellular components *via* autophagy 21. Both endosomes and lysosomes play an important role in endocytosis and degradation, thereby forming an endolysosomal system 22, 23.

### 2.1 ER network

**Figure 1:**
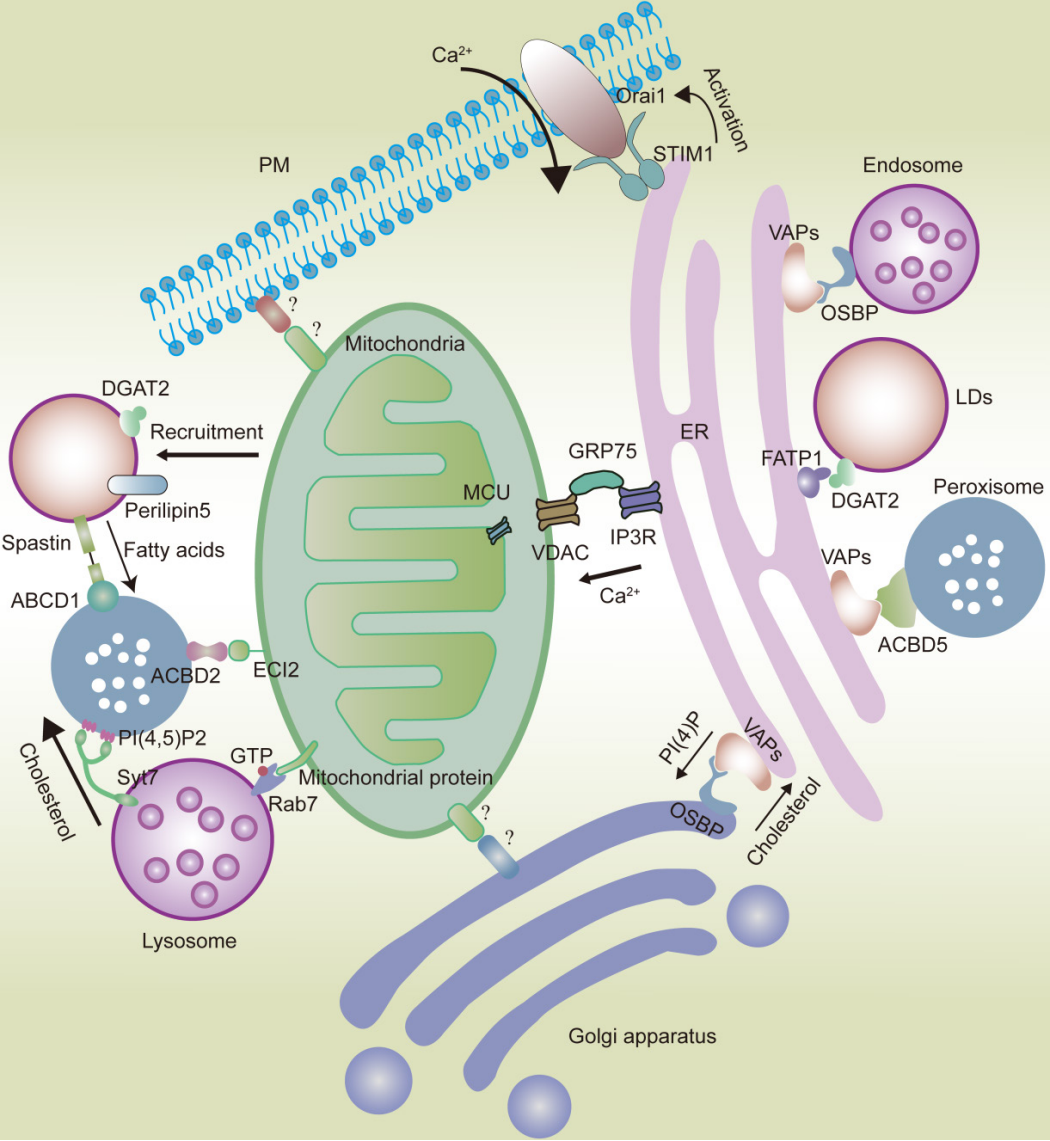
In eukaryotic cells, organelles widely interact with each other, forming networks such as the ER and mitochondrial networks. The ER interacts with the mitochondria *via* the IP3R-GRP75-VDAC-MCU complex. VAPs and OSBP play an important role in the interaction between the ER and the Golgi apparatus or endosomes. VAPs also play a significant role in ER-peroxisome interactions by coming in contact with ACBD5. FATP1 and DGAT2 bridge the ER to the LDs. The ER is also anchored to the PM by the STIM1-Orai1 interactions. Mitochondria interact with the Golgi apparatus and PM. However, the composition of mitochondria-Golgi apparatus and mitochondria-PM MCS tethers in mammalian cells remains unknown. ECI2/ACBD2 plays a significant role in the mitochondria-peroxisome MCSs. Rab7 plays an important role in the mitochondria-lysosome interaction. LDs can recruit mitochondria *via* DGAT2 and perilipin5. Syt7 in lysosomes interacts with PI(4,5)P2 in peroxisomes. Peroxisomes also come in contact with LDs via the spastin-ABCD1 interaction.

With extensive reticular structures 14, ER membranes can form abundant physical connections with the PM 24 and nearly all membrane-bound organelles 7, 25, such as mitochondria 26, 27, endosomes/lysosomes, phagosomes 28-32, Golgi apparatus 33, peroxisomes 34, and LDs 35. The ER provides structural support for the transport of membrane-bound organelles 8, but is also connected with membraneless organelles 25, 36, thereby forming an intracellular network hub (Fig.1).

The stability of MCS requires tethering proteins, such as vesicle-associated membrane protein-associated proteins (VAPs), which play a key role in maintaining ER stability 6, 8, 37. VAPs are highly conserved type II integral ER membrane proteins that can bind various intracellular proteins containing phenylalanine-phenylalanine acidic tract (FFAT) motifs 6, 38, 39.

Proteins containing FFAT motifs also have domains that can bind to lipids and other proteins 6. For example, the oxysterol-binding protein-related protein family (ORPs) contains FFAT motifs and can be found in endosomes and the Golgi apparatus 39, 40. VAPs can also bind to other proteins containing FFAT motifs, such as phosphatidylinositol transfer protein membrane associated 1 (PITPNM1, as known as Nir2) and ceramide-transfer protein (CERT) in the Golgi apparatus 41, 42, steroidogenic acute regulatory protein-related lipid transfer domain-3 (STARD3) and oxysterol-binding protein-related protein 1 (ORP1L) in endosomes 8, 43, 44, and acyl-CoA binding domain protein 5 (ACBD5) in peroxisomes 45. The orthologues of VAPs in plants and yeast have similar effects 39, 46.

#### 2.1.1 ER-PM interaction

##### 2.1.1.1 ER-PM MCSs and lipid transport

Most lipids are synthesized in the ER and then are distributed to other membranes 47. In mammalian cells, the ER and the PM form extensive MCSs, occupying approximately 2-5% of the cytoplasmic surface area 8. It has been found that the extended synaptotagmin-like proteins (E-Syts), VAPs, transmembrane (TMEM) family, ORP5, and ORP8 contribute to ER-PM tethering 2, 8 and non-vesicular lipid transport between the ER and the PM 8, 48. Notably, the absence of ER-PM tethers slows cell growth and reduces lipid content 47.

ER-PM MCSs play a key role in lipid transport. For example, ORP5 and ORP8 in the ER tether the ER to the PM through interactions with phosphatidylinositol 4-phosphate (PI(4)P) in the PM, thereby forming ER-PM MCSs to deliver PI(4)P to the ER for degradation and to deliver phosphatidylserine (PS) from the ER to the PM, to control PI(4)P levels and enrich PS in PM 8, 48, 49. The E-Syts SMP domain can dimerize to form a long hydrophobic tunnel, allowing phospholipids to move between the ER and PM membranes 8, 37. TMEM24 promotes ER-PM MCS formation to transfer phosphatidylinositol (PI) from the ER to the PM while decreasing Ca^2+^ concentration in the cytoplasm 8. Nir2 in PM can bind to VAPs in the ER to form MCS, thus delivering PI from the ER to the PM and palmitic acid from the PM to the ER 8, 37, 50.

##### 2.1.1.2 ER-PM MCSs and Ca^2+^ regulation

ER-PM MCSs also play a key role in Ca^2+^ regulation. The stromal interaction molecule 1 (STIM1)-Orai1 interaction in ER-PM MCSs plays an important role in calcium regulation 51, 52. STIM1 in the ER is a sensor of Ca^2+^ concentration, and it translocates to ER-PM MCSs following the depletion of stored Ca^2+^, to bind to and activate PM-located Orai1, a hexameric Ca^2+^ release-activated Ca^2+^ (CRAC) channel 8, 53-55. Orai1 mediates the influx of extracellular Ca^2+^, activates the sarco/ER Ca^2+^-ATPase (SERCA) pump, and guides Ca^2+^ into the ER lumen 8, 51. The interaction between ryanodine receptor 1 (RyR1) in the ER and Cav1.1, a subunit of the voltage-dependent calcium channel in the PM, forms ER-PM MSCs and plays a significant role in controlling Ca^2+^ flux to drive cell contraction 8, 51. When an action potential is generated in the PM, Cav1.1 undergoes a conformational change, leading to Ca^2+^ release *via* RyR1 into the cytoplasm 8, 56, which further induces cell contraction.

#### 2.1.2 ER-mitochondria interaction

##### 2.1.2.1 ER-mitochondria MCSs and lipid transport

The ER and the mitochondria can also form extensive MCSs, with a distance of approximately 10-25 nm, occupying approximately 2-5% of the mitochondrial surface area 8, 27. Mitofusin 2 (Mfn2) is a major contributor in the formation of ER-mitochondria MCSs. ER-mitochondria MCSs are reduced by 40% when cells lack Mfn2 27. There are many protein interactions in ER-mitochondria MCSs, including ORP, tetrameric inositol 1,4,5-triphosphate receptor (IP3R), and B‐cell receptor‐associated protein 31 (BAP31) in the ER, protein tyrosine phosphatase interacting protein 51 (PTPIP51), voltage-dependent anion-selective channel protein (VDAC), and mitochondrial fission 1 (FIS1) in the mitochondria, and glucose‐regulated protein 75 (GRP75) in the cytoplasm 48, 57.

Bidirectional lipid transfer between the mitochondria and the ER plays an important role in maintaining the defined lipid composition of mitochondrial membranes 57. Since the mitochondria cannot synthesize PS, it must be imported from the ER 57. PS is transferred from the ER to the mitochondria by ORP5 and ORP8 interaction with PTPIP51 in ER-mitochondria MCSs 48. After PS is converted into phosphatidylethanolamine (PE) in the mitochondria, PE is transferred from the mitochondria to the ER 57-59. However, the role of ER-mitochondria MCSs in PE export from the mitochondria is unclear 58.

##### 2.1.2.2 ER-mitochondria MCSs and Ca^2+^ regulation

ER-mitochondria MCSs can transfer Ca^2+^ from the ER lumen to the mitochondrial matrix 8, 60, 61. The IP3R-GRP75-VDAC-mitochondrial calcium uniporter (MCU) complex is required in this process (a more detailed description is provided in Section 3.1.1) 62. Other proteins are also important for ER-mitochondria MCSs. For instance, PDZ domain‐containing protein 8 (PDZD8), a protein found concentrated in ER-mitochondria MCSs, is necessary for ER-mitochondria MCS formation in mammalian cells and is required for Ca^2+^ uptake by the mitochondria after calcium release from the ER 63. Mfn2 ablation leads to the absence of ER-mitochondria MCSs, which reduces the efficiency of mitochondrial Ca^2+^ uptake 27, 64. PTPIP51 also plays an important role in Ca^2+^ uptake of the mitochondria from the ER, by binding to vesicle-associated membrane protein-associated protein B (VAPB) 65. Moreover, it has been shown that Ca^2+^ transfer from the mitochondria to the ER occurs via an unidentified Na^+^/Ca^2+^ exchanger 66.

##### 2.1.2.3 ER-mitochondria MCSs and mitochondrial dynamics

Mitochondria are dynamic organelles that maintain proper function, quality, and distribution through continuous fission and fusion 8, 67. The regulatory mechanisms of the ER involved in mitochondrial dynamics *via* ER-mitochondria MCSs are described in detail in Section 3.3.2.

##### 2.1.2.4 ER stress and unfolded protein response (UPR)

A more detailed description of ER stress and UPR is provided in Section 3.2.1.

##### 2.1.2.5 Autophagosome formation

A more detailed description of autophagosome formation is provided in Section 3.2.2.

#### 2.1.3 ER-Golgi apparatus interaction

The bidirectional vesicular trafficking between the ER and the Golgi apparatus is not the only mechanism of interaction between the two organelles. Previous studies have reported that non-vesicular transport, which mainly involves the transfer of lipids, also plays an important role in the interaction between the two organelles 33. Vesicular trafficking mainly occurs on the nuclear side of the Golgi apparatus, while ER-Golgi apparatus MCSs are mainly located on the cytomembrane side of the Golgi apparatus, with a distance of approximately 10-20 nm between the ER and the Golgi apparatus 33.

In the ER-Golgi apparatus MCSs, ER protein VAPs bind to Nir2, CERT, and oxysterol-binding protein (OSBP) in the Golgi apparatus, all of which have FFAT motifs and play an important role in lipid transfer between the ER and the Golgi apparatus 8. Nir2 transfers PI from the ER to the Golgi apparatus, decreasing PI concentration in the ER. Furthermore, PI is converted into PI(4)P, recruiting CERT and OSBP to ER-Golgi MCSs by binding to their PH domains 41. CERT is responsible for the transport of ceramide between the ER (the site of its synthesis) and the Golgi apparatus (the site of its conversion to sphingomyelin) 33. In addition, OSBP plays a significant role in the exchange of PI(4)P for cholesterol. In ER-Golgi apparatus MCSs, PI(4)P levels are low because of the ER phosphatase Sac1, which converts PI(4)P into PI. PI(4)P is transferred from the ER to the Golgi apparatus to form a concentration gradient which drives cholesterol transport 8, 33, 68, 69.

#### 2.1.4 ER-peroxisomes interaction

Peroxisomes, originating from the division of existing peroxisomes or from the ER 1, 70, 71, play an essential role in lipid synthesis, breakdown and detoxification of fatty acids, and detoxification of reactive oxygen species (ROS) 8, 72. To mediate these functions, peroxisomes interact with other organelles *via* vesicular trafficking or MCSs 72. The interaction between peroxisomes and the ER is important for the biosynthesis of sterols, unsaturated fatty acids, and ether‐phospholipids 72. For instance, the synthesis of ether-linked phospholipids is initiated in peroxisomes, but is completed in the ER 8.

Peroxisomes lack an appropriate enzyme inventory for autonomous lipid synthesis. Phospholipids essential for the growth and division of peroxisomes are provided by the ER 8, 73. Lipid transfer between peroxisomes and the ER depends on ER-peroxisome MCSs rather than on vesicles 73. Notably, the interaction between ACBD5 in peroxisomes and VAPs in the ER plays an important role in maintaining the ER-peroxisome MCSs and lipid transfer 74. The overexpression of VAPs or ACBD5 increases the number and surface area of ER-peroxisome MCSs 8. Moreover, the interaction between phosphatidylinositol‐4, 5‐bisphosphate (PI(4,5)P2) in peroxisomes and E-Syts in the ER plays an important role in cholesterol transfer from peroxisomes to the ER 75. In addition, ER-peroxisome MCSs play a role in peroxisome mobility and distribution. The disruption of ER-peroxisome MCSs has been shown to increase the number and displacement of moving peroxisomes 8, 73.

#### 2.1.5 ER-LD interaction

A more detailed description is provided in Section 3.4.

#### 2.1.6 ER-endolysosomal organelle interaction

Both endosomes and lysosomes play an important role in the degradation of the cargo of cellular endocytosis, thereby forming an endolysosomal system 22, 23. The ER-endolysosomal organelle MCSs regulate certain processes in these organelles 23. The numbers of contacts between endosomes and the ER increase as the endosome matures. Approximately 50% of early endosomes and more than 99% of late endosomes are in contact with the ER, and their MCSs occupy approximately 5% of the endosome surface area.

Intracellular cholesterol mainly originates from endocytosis of low-density lipoprotein (LDL). LDL in early endosomes is delivered to the late endosomes/lysosomes and then hydrolyzed and transported by vesicular or non-vesicular mechanisms to downstream organelles, including the PM, ER, and mitochondria, to perform its functions 76. Approximately 30% of LDLs from endosomes reach the ER *via* MCSs 23, 76. VAPs and endosome-localized proteins, STARD3, ORP1L, and OSBP, play key roles in maintaining ER-endosome MCSs and regulating lipid transport. The interaction between VAPs and the late endosome cholesterol-binding protein ORP1L promotes cholesterol transport from the late endosomes to the ER. However, STARD3, which can bind cholesterol and transfer it between membranes, plays the opposite role 23. VAP-OSBP interaction contributes to phospholipid homeostasis in ER-endosome MCSs by recruiting PI(4)P-phosphatase Sac1 to MCSs to further decrease PI(4)P level in endosomes 8, 23, 77. A previous report has shown that high levels of PI(4)P block cargo trafficking from endosomes to the Golgi apparatus 8.

ER-endolysosomal organelle MCSs also play an important role in endolysosomal fission and positioning 23. It has been reported that endosomal tubule fission occurs at ER-endosome MCSs in approximately 80% of cases 78. The interaction between endosome-located IST1 factor associated with the endosomal sorting complex required for transport-III (IST1) and ER-located spastin links endosomes to the ER and regulates endosome fission 78. Loss of spastin leads to impaired endosomal tubule fission and abnormal lysosomal morphology 23. In addition, when cholesterol levels are high, ORP1L promotes endosome trafficking to the cell center, where they can fuse with lysosomes. However, when cholesterol levels are low, ORP1L leads to the accumulation of late endosomes at the cell periphery 23. It has also been found that ER-endolysosomal organelle MCSs result in Ca^2+^ uptake from the ER. For example, IP3Rs have been found to be clustered at ER-lysosome MCSs and deliver Ca^2+^ to lysosomes 23.

### 2.2 Mitochondrion network

Mitochondria are the primary sites of ATP production. Therefore, mitochondrial homeostasis plays a crucial role in a variety of biological processes 79. Mitochondria interact with other organelles to maintain cellular homeostasis. Mitochondria have extensive contact with PM 80 and other organelles 31, 57, such as the LDs 81, Golgi apparatus 82, lysosomes 83-85, and peroxisomes 86. As a result, these organelles form a secondary intracellular reticulum network that acts as an important cellular signaling platform 31, 86.

#### 2.2.1 Mitochondria-PM interaction

PM-mitochondria MCSs are better studied in the processes involved in mitochondrial fission and Ca^2+^ uptake 31. Almost 10% of the PM is adjacent to mitochondria in animal cells 87. Notably, PM-mitochondria MCSs also play a role in the distribution of the mitochondria during cell division 80, 87. Subplasmalemmal mitochondria relay Ca^2+^ to the ER by modulating the activity of Ca^2+^-ATPases in the PM 88.

#### 2.2.2 Mitochondria-lysosome interaction

Mitochondria and lysosomes are critical organelles involved in cellular homeostasis. Studies have shown that mitochondrial respiration dysfunction leads to lysosomal defects 89. Lysosomes can regulate mitochondrial functions in a peroxisome proliferator-activated receptor gamma coactivator 1-alpha (PGC-1α)-independent manner through the biosynthesis of transcription factor EB (TFEB). Moreover, the dysfunction of lysosomal acidification leads to decreased mitochondrial respiration 85. Mitochondria and lysosomes can also directly interact under stress conditions *via* mitochondrial-derived vesicles (MDVs) and mitophagy 85.

Mitochondria-lysosome MCSs are abundant and can be identified by electron microscopy in healthy cells 84. Unlike MDVs fusing with lysosomes or mitophagy, mitochondria-lysosome MCSs do not result in bulk transfer of mitochondrial luminal content or mitochondrial degradation 84. Mitochondria-lysosome MCSs have an average distance of 10 nm between the membranes and play an important role in modulating the dynamics of both organelles and ensuring Ca^2+^ transfer. Rab7, a GTPase, is an important protein that modulates the dynamics of mitochondria-lysosome MCS tethering and untethering 85. Rab7 exhibits an active GTP-bound state under normal conditions, but shows an inactive GDP-bound state upon GTP hydrolysis 84. The interaction between GTP-Rab7 in lysosomes and Rab7 effectors in the mitochondria plays a central role in maintaining the stabilization of mitochondria-lysosome MCSs. However, FIS1, the outer mitochondrial membrane (OMM) protein, recruits cytosolic TBC domain family member 15 (TBC1D15) to drive GTP-bound Rab7 hydrolysis to a GDP-bound state, thereby leading to untethering of MCSs 85.

Mitochondria can also modulate lysosomal retrograde and anterograde microtubule transport and coordinate lysosome dynamics *via* mitochondria-lysosome MCSs 84, 85. These MCSs can also regulate mitochondrial dynamics (details are provided in Section 3.2.2). Mitochondria-lysosome MCSs also play a role in Ca^2+^ uptake by the mitochondria. The lysosomal channel transient receptor potential mucolipin 1 (TRPML1)-VDAC-MCU complex plays a significant role in the direct transfer of calcium from lysosomes to the mitochondria *via* mitochondria-lysosome MCSs 90. The activation of TRPML1, a calcium-releasing channel on the lysosomal membrane, can increase the concentration of Ca^2+^ in mitochondria-lysosome MCSs 85, 90. Furthermore, Ca^2+^ enters the mitochondrial matrix *via* the VDAC channel in the OMM and MCU channels in the inner mitochondrial membrane (IMM) 90. TRPML1 activity is increased due to increased ROS 85.

#### 2.2.3 Mitochondria-peroxisome interaction

Both mitochondria and peroxisomes play important roles in lipid and ROS metabolism 86, suggesting that there may be an interaction between these two organelles to balance this process. Peroxisomes and mitochondria exhibit a close functional interaction, including metabolic cooperation in regulating fatty acid β-oxidation and maintaining ROS homeostasis, sharing a redox-sensitive relationship, and cooperation in viral combat 91. Mitochondria and peroxisomes interact with each other *via* MCSs, vesicular traffic, and biological messengers, such as ROS, lipids, and other metabolites 86, 91. In yeast, Pex11p is required for mitochondria-peroxisome MCS formation 31. Peroxisome-located acyl-CoA binding domain protein 2 (ACBD2) and mitochondria-located enoyl-CoA-δ isomerase 2 (ECI2) play an important role in the formation of mitochondria-peroxisome MCSs and steroids in mammals 92. These two organelles also share key components of their division machinery in MCSs, including dynamin-related protein-1 (DRP1), fission factor 1, mitochondrial fission factor, and ganglioside-induced differentiation-associated protein 1 91.

Mitochondrial function is impaired in senescent cells, and this can be compensated by peroxisomes *via* the mitochondrial retrograde (RTG) signaling pathway 93. Following the increasing transcription of mitochondrial and peroxisomal genes related to carbohydrate and nitrogen metabolism, peroxisome proliferation is activated and fatty acids are converted into acetyl-CoA to replenish the tricarboxylic acid (TCA) cycle in mitochondria 93, 94.

#### 2.2.4 Mitochondria-LD interaction

A more detailed description is provided in Section 3.4.

### 2.3 Other organelle interactions

There are other organelle interactions, including peroxisome-lysosome 95, 96 and peroxisome-LD interactions 97. Although the interactions of peroxisomes with lysosomes appears to be less frequent than their interaction with the ER or the mitochondria, peroxisome-lysosome interactions are still found in approximately 15% to 20% of the total peroxisomes in mammalian cells 73. The lysosome-peroxisome MCSs are the principal pathway for cholesterol transfer from the lysosome to the peroxisome 76. Synaptotagmin-7 (Syt7), on lysosomes, and PI(4,5)P2, on peroxisomes, form peroxisome-lysosome MCSs, which play an important role in cholesterol transport 34, 73, 76. When cholesterol reaches peroxisomes, it participates in peroxisome biogenesis 76. The disruption of lysosome-peroxisome MCSs decreases cholesterol levels in the PM and impairs cholesterol transport to the ER, suggesting that lysosome-peroxisome MCSs may be associated with cholesterol transport to other organelles 76. MCSs may also regulate other functions of lysosomes and peroxisomes, such as autophagy and peroxisome biogenesis 76. A more detailed description of LD-peroxisome interactions is provided in Section 3.4.

## 3. The role of organelle interaction in cellular senescence

Cellular senescence is a permanent cell cycle arrest induced by a series of stress factors, including telomere attrition, DNA damage, oxidative stress, oncogene activation, and organelle stress. Cellular senescence is characterized by chromatin remodeling, metabolic reprogramming, and senescence-associated secretory phenotype (SASP) 10, 98-101. Senescent cells accumulate in organs during aging and secrete SASP molecules, including proinflammatory and fibrogenic molecules, to further promote a variety of age-related diseases 101, 102. Senescence can be classified into the following types: replicative, DNA-damage-induced, stress-induced, and oncogene-induced 103.

### 3.1 Cellular calcium homeostasis and senescence

#### 3.1.1 ER and mitochondrial calcium homeostasis

**Figure 2:**
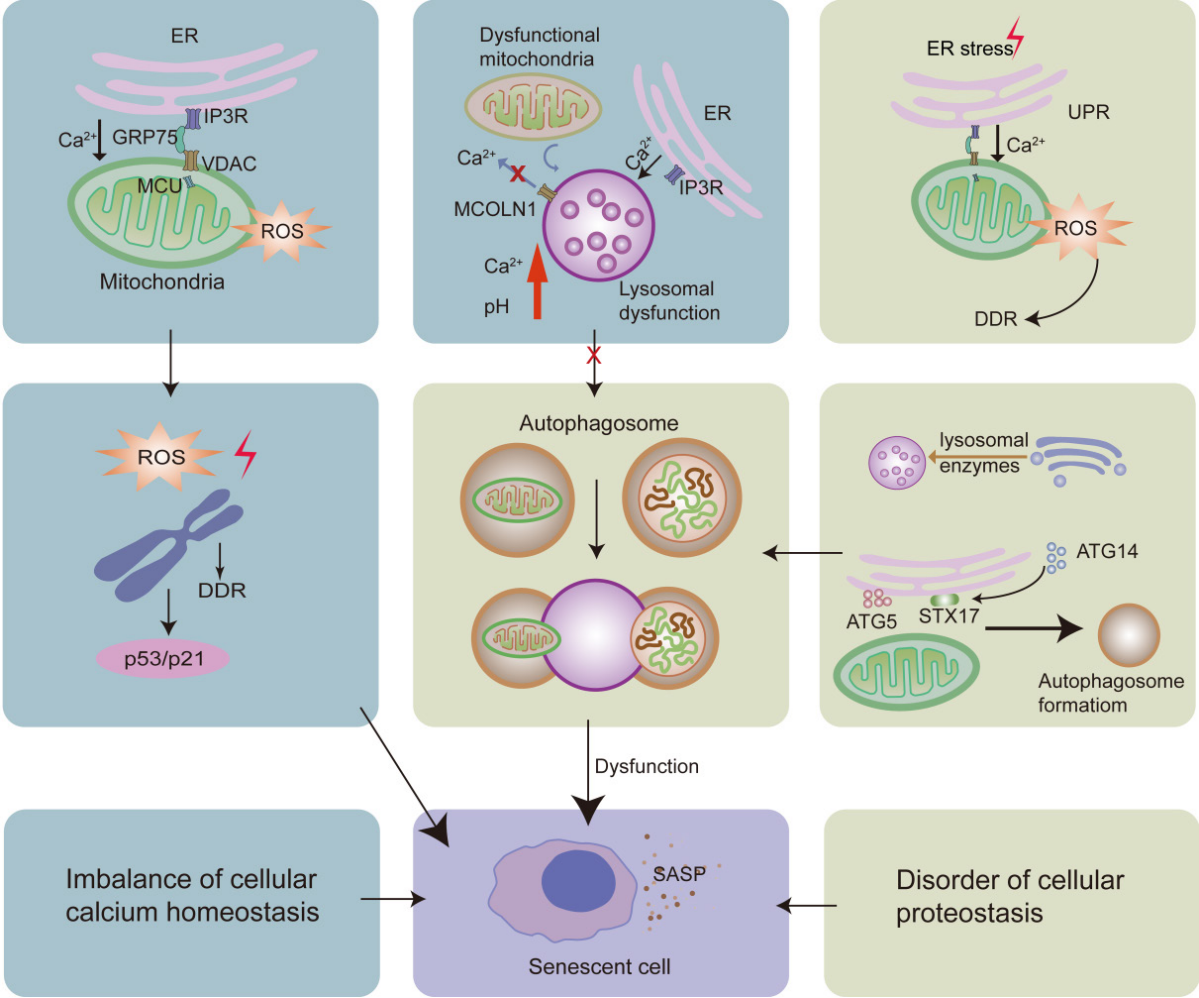
The increased calcium released from the ER can be transferred to the mitochondria* via* the IP3R-GRP75-VDAC-MCU complex. This leads to the increased levels of ROS in the mitochondria, contributing to DNA damage and DDR, activating the p53/p21 pathway, and causing cellular senescence. Moreover, while the UPR is consistently activated, it also leads to calcium accumulation in the mitochondria, causing cellular senescence. On the one hand, dysfunctional mitochondria can repress the lysosomal calcium-releasing channel MCOLN1; on the other hand, there is increased calcium release from the ER *via* IP3R. Both these processes lead to calcium accumulation and an increase in pH in the lysosomes, resulting in dysfunction of the lysosome-autophagy proteolytic system and contributing to cellular senescence. Lysosomes can originate from the direct acidification of late endosomes. The Golgi apparatus deliver lysosomal enzymes to endosomes. Moreover, ER-mitochondria MCSs promote the formation of autophagosomes. Impaired autophagosomal formation and lysosomal function leads to the dysfunctional autophagy, thereby causing cellular senescence.

The ER is a dynamic calcium reservoir that maintains cellular calcium homeostasis 104. In senescent cells, calcium is released from the ER to increase intracellular calcium levels 105. Mitochondria and lysosomes also play crucial roles in cellular calcium homeostasis 85. Hence, cellular calcium buffering is controlled by the interplay between ER, mitochondria, and lysosomes 106. Studies have shown that an imbalance in ER calcium homeostasis not only induces the production of SASP molecules, but also causes cell cycle arrest by influencing mitochondrial calcium homeostasis 105. In particular, appropriate calcium transfer from the ER to the mitochondria requires the correct distance between the two organelles 107 and accurate organelle interaction. ER-mitochondria MCS dysfunction can be found in senescent cells, which further leads to an imbalance in calcium control 108-110. ER-mitochondria MCSs play an important role in Ca^2+^ homeostasis *via* the IP3R-GRP75-VDAC-MCU complex. Ca^2+^ is released through the IP3R channel in the ER, which then enters the mitochondrial matrix *via* the VDAC channel in the OMM and MCU channels in the IMM 8, 111. The VDAC channel is crucial for mitochondrial Ca^2+^ uptake 90, and the MCU channel is considered to be the major transporter of Ca^2+^ into the mitochondrial matrix 90. Notably, GRP75 plays a role in linking IP3R and VDAC to shorten the distance between the ER and the mitochondria 62. In cellular senescence, IP3R-mediated calcium release from the ER is activated and the expression of MCU increases 105, 112, leading to the accumulation of mitochondrial calcium and contributing to a decrease in mitochondrial membrane potential and increased ROS production 105. Excess ROS can induce DNA damage and lead to single-strand breaks in telomere regions, resulting in accelerated telomere shortening, both of which can activate the DNA damage response (DDR) pathway 100, 109, 113, 114. Consequently, DDR performs cell cycle checkpoint functions and blocks cell cycle progression. After DNA damage, DDR factors accumulate at the sites of DNA damage and cause chromatin modifications, such as the phosphorylation of histone H2AX. Persistent DNA damage causes a prolonged DDR signaling pathway. It induces the activation of the tumor suppressor p53, contributing to the expression of the cyclin-dependent kinase inhibitor p21, to cooperatively induce cellular senescence 100. It has been shown that IP3R knockdown can attenuate cellular senescence by reducing the number of ER-mitochondria MCSs and ER calcium release 115, 116. Moreover, MCU knockdown can also prevent cellular senescence by attenuating mitochondrial calcium intake 117 (Fig.2).

#### 3.1.2 Lysosomal calcium homeostasis

Lysosomal calcium homeostasis plays an important role in maintaining lysosomal function. In senescent cells, the activated IP3R in the ER leads to increased ER calcium release 105 *via* ER-lysosome MCSs, resulting in lysosomal calcium accumulation. The imbalance of lysosomal calcium homeostasis induces disorders related to lysosomal acidification 118, which are associated with lysosomal proteolytic activity 119. Furthermore, impaired proteolytic activity of lysosomes negatively affects the lysosome-mediated autophagy proteolytic system and causes cellular senescence 102, 120-123.

In senescent cells, damaged mitochondria accumulate 124. Dysfunctional mitochondrial respiratory chain impairs lysosomal calcium homeostasis by repressing the lysosomal calcium-releasing channel mucolipin 1 (MCOLN1, also known as TRPML1) 125, plays an important role in regulating lysosomal biogenesis, exocytosis, and autophagy *via* the regulation of lysosomal calcium release 126. The impairment of the function of TRPML1 leads to enlarged lysosomes, lysosomal calcium accumulation, and increased pH, which further contributes to lysosomal malfunction and cellular senescence 119, 126. It has been reported that mitochondrial ROS can activate TRPML1, inducing lysosomal calcium release 85, further inducing autophagy and lysosomal biogenesis 127 and delaying cellular senescence. However, excess ROS levels in the mitochondria have the opposite effect, leading to lysosomal dysfunction and cellular senescence 126, 127.

Transmembrane BAX inhibitor motif containing 6 (TMBIM6), a calcium channel-like protein integral to the intracellular membranes of the ER, can also regulate lysosomal calcium 128. Specifically, it increases lysosomal calcium release under stress conditions to restore cellular homeostasis by enhancing lysosomal calcium release-induced autophagy 128, thereby retarding cellular senescence 129.

### 3.2 Cellular protein homeostasis and senescence

Cellular protein homeostasis is crucial for maintaining cellular function. Dysregulation of cellular protein homeostasis contributes to cellular senescence and aging 130-134. It has been shown that cellular proteostasis is decreased in senescent cells 135. The imbalance of cellular protein homeostasis plays an important role in senescence-related pathologies 102, while improved proteostasis can delay aging 133. Under persistent stress, cellular proteins are damaged, aggregated, misfolded, or no longer needed. The three primary pathways that are used by senescent cells to maintain cellular protein homeostasis are as follows: utilizing molecular chaperones, the proteasome proteolytic system, and the lysosome-autophagy proteolytic system 130, 133, 134, 136.

#### 3.2.1 UPR and cellular senescence

Most secretory proteins in eukaryotic cells undergo chaperone-assisted folding in the ER to acquire an appropriate spatial structure 137. When cells are under stress, unfolded or misfolded proteins accumulate in the ER lumen, contributing to ER stress 137. To maintain ER function and cellular protein homeostasis, the UPR is activated by the ER-localized transmembrane stress sensors, namely inositol requiring enzyme 1 alpha (IRE1α), RNA-dependent protein kinase-like ER kinase (PERK), and activating transcription factor 6 alpha (ATF6α) 66, 137. Both IRE1α and ATF6α play important roles in enhancing the degradation of misfolded proteins. Under ER stress, full-length ATF6 in the ER translocates to the Golgi apparatus, where it is cleaved into ATF6p50; then, it transitions into the nucleus to induce gene expression. PERK can induce a transient decrease in protein synthesis to relieve ER stress through the attenuated influx of newly synthesized proteins into the ER lumen 137.

During the early phases of ER stress, the UPR boosts pro-survival signaling 66, which increases and tightens the ER-mitochondria MCSs and increases mitochondrial Ca^2+^ uptake, thereby driving adaptive mitochondrial metabolic boost and ATP production to establish the metabolic basis for recovering cell homeostasis 57, 107, 138. However, when stress is not resolved, the UPR leads to excess intake of mitochondrial Ca^2+^, causing cellular senescence or even cell death 99, 139, 140. UPR activation occurs in almost all types of senescence 99. The UPR is not only a consequence of cellular senescence, but also its driver 99. However, the mechanism by which UPR induces senescence remains poorly understood.

It has been reported that a variety of ER chaperones involved in protein folding are enriched in ER-mitochondria MCSs 141, such as PERK and IRE1α 137. PERK can facilitate the tethering of the ER to the mitochondria, leading to increased mitochondrial calcium intake and ROS production under ER stress 137. Sustained ER stress leads to excess release of Ca^2+^ from the ER *via* IP3R. Calcium is enriched in ER-mitochondria MCSs and is taken up by the mitochondria *via* calcium uniporter. Large calcium influx leads to the loss of the mitochondrial membrane potential. Furthermore, cytochrome c translocates into the ER-mitochondria MCSs and binds to IP3R, which abolishes the calcium-mediated inhibition of IP3-associated calcium release and results in a feed-forward amplification of ER calcium release 66. Finally, this causes an imbalance in mitochondrial Ca^2+^ homeostasis 139, 140 and increases ROS production 105, 142, thereby resulting in cellular senescence 109, 143.

#### 3.2.2 Lysosome-autophagy proteolytic system and cellular senescence

Autophagy is a process of self-degradation of organelles and misfolded proteins by autophagosomes, which are then delivered to lysosomes 144. The lysosome-autophagy proteolytic system plays a significant role in maintaining cellular protein homeostasis, and its imbalance contributes to cellular senescence 134, 145. It has been reported that autophagy activation can prevent cellular senescence by downregulating p53 phosphorylation and p21 levels 146. There are three main types of autophagy in mammalian cells that maintain the homeostasis of proteins and organelles: microautophagy, macroautophagy, and chaperone-mediated autophagy 145. Lysosomal-mediated autophagosomal degradation plays an important role in maintaining cellular homeostasis under stress conditions 119, 128, 131. Damaged proteins and organelles can induce cellular senescence 147. Notably, autophagy plays a significant role in their degradation, providing adequate amino acids and maintaining energy levels to support cells 147-149. Autophagy inhibition and insufficiency lead to cellular senescence 145, while increased autophagy delays aging 123, 150.

The formation of autophagosomes is the first step in the macroautophagy 148. ER-mitochondria MCSs are the origins of autophagosomes 151. When autophagy signals initiate, autophagosome formation-related proteins accumulate on the ER side of the ER-mitochondria MCSs 57, 152. Autophagy-related gene 14 (ATG14), the pre-autophagosome marker, and autophagy-related gene 5 (ATG5), the autophagosome-formation marker, are enriched in ER-mitochondria MCSs after starvation. ATG14 is recruited to ER-mitochondria MCSs by syntaxin 17 (STX17), an ER-resident protein 151. Mitochondria also supply membranes for autophagosome biogenesis 153.

Lysosomes play an important role in the final step of the lysosome-autophagy proteolytic system by fusing with autophagosomes. Lysosomal function affects the lysosome-autophagy proteolytic system. In senescent cells, lipofuscin accumulates in lysosomes, leading to diminished lysosomal activity 154. In contrast, the activation of lysosomes restores the stemness of senescent stem cells 155. Lysosomes can originate from the direct acidification of late endosomes 21. In this process, lysosomal enzymes are transported from the Golgi apparatus to endosomes 156. Previous studies have shown that IST1-spastin interaction in ER-endosome MCSs plays an important role in endosomal tubule fission and in the transfer of lysosomal enzymes from the Golgi apparatus to endosomes 78. Lack of spastin or IST1 leads to lysosomal defects and abnormalities 78.

### 3.3 Mitochondrial quality control (MQC) and senescence

Mitochondrial dysfunction plays a crucial role in cellular senescence 109, 157. Cellular senescence accelerates mitochondrial dysfunction and, conjointly, mitochondrial dysfunction is also a cause of cellular senescence 124, 158, 159. Increased mitochondrial ROS production induces DNA damage, activates DDR, and contributes to cellular senescence. Furthermore, decreased NAD^+^/NADH ratio in dysfunctional mitochondria leads to the persistent activation of the energy sensor AMP-activated protein kinase (AMPK), which induces cellular senescence by phosphorylating p53 and stabilizing p16^INK4a^ mRNA 159, 160.

**Figure 3:**
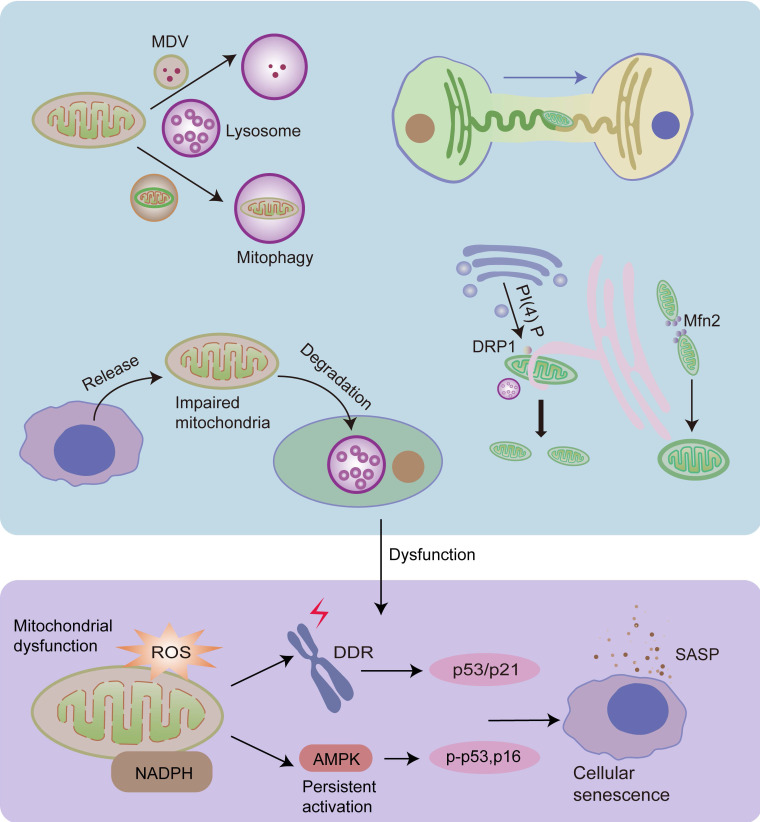
Mitochondrial fission and fusion, mitophagy, MDVs, and mitochondrial transfer play important roles in MQC, and they are regulated by organelle interactions. Damaged mitochondrial proteins can be transported into lysosomes in the form of MDVs, and dysfunctional mitochondria can be degraded by lysosomes* via* mitophagy. Mitochondria can preserve shape and reduce damage *via* continuous fusion and fission cycles. ER tubules physically wrap around and constrict the mitochondria, promoting mitochondrial fission. Moreover, Golgi-derived vesicles containing PI(4)P are necessary for this process. Mitochondria-lysosome interactions also play an important role in mitochondrial fission. In addition, ER regulates mitochondrial fusion, and Mfn2 plays a significant role in this process. Dysfunctional mitochondria can also be released from cells and degraded by lysosomes of other cells. Furthermore, healthy cells can transfer healthy mitochondria into other cells *via* the ER tuber. Dysfunctional MQC leads to the accumulation of impaired mitochondria, leading to increased ROS levels and decreased NAD^+^/NADH ratios, both of which contribute to cellular senescence.

MQC is an elaborate mechanism that maintains normal mitochondrial function. It includes two opposite processes: mitochondrial biogenesis and the removal of impaired mitochondria or their components 161. Dysregulation of MQC induces the accumulation of damaged mitochondria, leading to cellular senescence 102. Damaged proteins can be transported into lysosomes or peroxisomes in the form of mitochondrial-derived vesicles (MDVs) as the first defense against mitochondrial damage 102, 162. The cargo delivered to lysosomes is ultimately degraded, however, the fate of the cargo delivered to peroxisomes is unclear 162. When mitochondria are depolarized or dysfunctional, they are removed by mitophagy 102. Cells can release damaged mitochondria into the extracellular space to maintain mitochondrial homeostasis 163, 164. In addition, cells can acquire mitochondria *via* mitochondrial fission or by receiving extracellular healthy mitochondria 161, 165 (Fig.3).

#### 3.3.1 MDVs and mitophagy

Lysosomes play an important role in the final degradation of damaged proteins in MDVs and dysfunctional mitochondria during mitophagy 166. Mitochondria-lysosome interactions play an important role in MQC 166. Mitophagy plays a role in the removal of dysfunctional mitochondria. The removed mitochondria are eventually replenished *via* biogenesis 166. Damaged mitochondrial components can also be degraded in the form of MDVs 166. Dysregulation of mitophagy leads to the accumulation of damaged mitochondria, thus contributing to cellular senescence 166.

However, in senescent cells, dysfunctional mitochondria produce high levels of ROS, which contribute to the accumulation of lipofuscin in lysosomes and lysosomal dysfunction 154. Moreover, damaged mitochondria also impair the calcium release of lysosomes, which further contributes to lysosomal dysfunction. Dysfunctional lysosomes cause impaired mitophagy and the function of degrading damaged mitochondrial proteins, leading to further mitochondrial malfunction. This feedback loop aggravates cellular senescence 154.

#### 3.3.2 Mitochondrial dynamics

Mitochondria can preserve homeostasis *via* continuous fusion and fission cycles 166. However, this process is impaired in senescent cells 109. It has been reported that mitochondrial dynamics involve the interaction of multiple organelles, including the Golgi apparatus, ER, and lysosomes.

ER-mitochondria MCSs have been shown to play an important role in mitochondrial fission and fusion by providing a series of enzyme-like nodes 39, 57, 167. Active mtDNA synthesis occurs at ER-mitochondria MCSs, and constriction of IMM is a priming event 67, 168. This suggests that the initial mitochondrial fission signal comes from the mitochondrial matrix, which then recruits the ER 8. During mitochondrial fission, ER tubules physically wrap around the mitochondria and lead to mitochondria constriction, facilitating the recruitment and assembly of DRP1, the main driver of mitochondrial fission 169. DRP1 then moves from the cytoplasm to the OMM at MCSs, constricting mitochondria down to less than 50 nm, and finally, Dnm2, a dynamin protein, is recruited to MCSs to induce fission 8, 57, 169-171. Furthermore, multiple studies have shown that Golgi-derived PI(4)P-containing vesicles are recruited to mitochondria-ER MCSs and can activate DRP1-mediated mitochondrial division. Disruption of PI(4)P production leads to the prevention of mitochondrial fission, and mitochondrial morphological defects 172. Mitochondria-lysosome MCSs also play a significant role in mitochondrial fission. It has been shown that more than 80% of mitochondrial fission events are marked by the lysosomal markers lysosomal associated membrane protein 1 (LAMP1)-positive vesicles 84, 85, 173. A study has also found that mitochondria-lysosome contacts play a role in ER-mediated mitochondrial fission 84. ER-mitochondria MCSs play an important role in mitochondrial fusion 57, 174, but the underlying mechanism is still unclear. Mfn2 may play a significant role in this process, as Mfn2 not only plays a critical role in mitochondrial fusion, but also in the maintenance of ER-mitochondria MCSs 64, 174, 175.

#### 3.3.3 Mitochondrial transfer

A novel MQC pathway involves the transfer of damaged mitochondria into the extracellular space for degradation 163. For example, the mitochondria released from neurons can be degraded by lysosomes of neighboring glial cells 176. This pathway is independent of mitophagy, and is activated by mitochondrial stress. When mitophagy is compromised, more mitochondria are released to compensate for the accumulation of damaged mitochondria 163.

Furthermore, intercellular mitochondrial transfer is a significant part of the MQC to rescue mitochondrial defects 163, 165. It has been reported that donor cells, especially stem cells, can transfer healthy mitochondria into recipient cells, leading to the improvement of mitochondrial bioenergetics and reduction of mitochondrial ROS 165, 177. There are four main methods for intercellular mitochondrial transfer, including utilizing tunneling nanotubes (TNTs), dendrites and microvesicles, and extrusion and internalization 165.

It has been shown that the mitochondrial transfer between osteocytes relies on the ER-mitochondria interaction 165. The inhibition of ER-mitochondria tethering significantly inhibits the transfer of the mitochondria between osteocytes 178. It has been hypothesized that the mitochondria are transferred along with the ER tuber into the terminus of osteocyte dendrites, where the ER contacts the PM. The arrival of mitochondria changes the lipid metabolism of the PM, which enables the ER of the donor to fuse with the ER extension of the recipient. Mitochondria are then transferred to the recipient along the fusing ER tubular network 178.

### 3.4 Lipid homeostasis and senescence

LDs play important roles in the maintenance of lipid homeostasis by coordinating lipid synthesis, storage, and secretion and lipolysis 179. Under fed conditions, free fatty acids are converted into neutral lipids by ER, and then deposited in LDs 93, 180. These neutral lipids include, but are not limited to, triacylglycerol, sterol esters, and esterified ceramides 179. Under the stress conditions or energy insufficiency, lipases on the surface of LDs can convert triglycerides into fatty acids, which are then imported into the mitochondria for β-oxidation to satisfy energy demands 31, 81, 181, 182.

**Figure 4:**
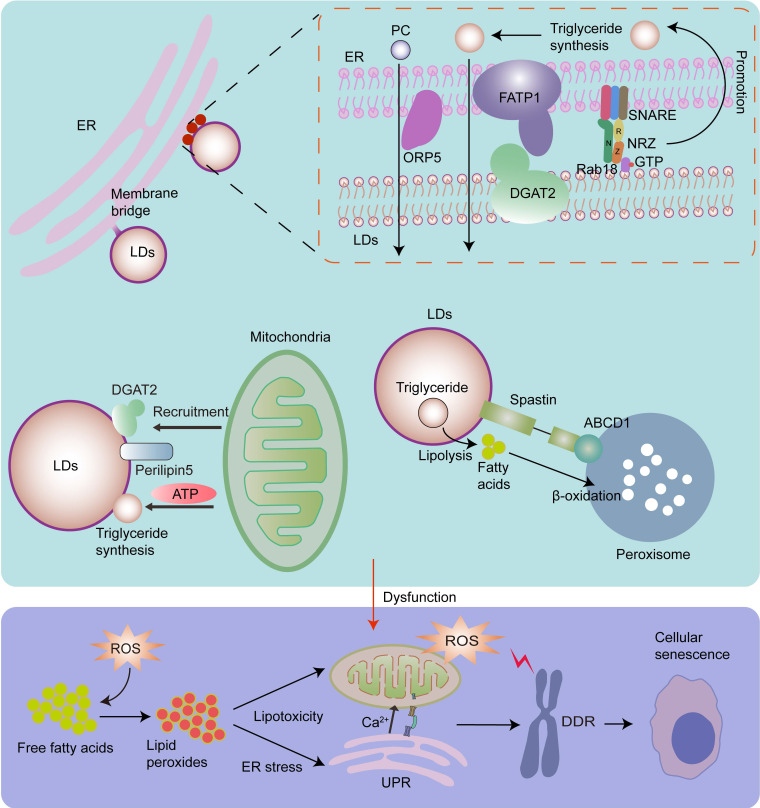
LDs originate from the ER, and the ER can transport triacylglycerol synthesis enzymes required for LD growth and expansion *via* membrane bridges. Rab18-NRZ-SNARE interaction facilitates LD growth and maturation by regulating the synthesis of triglycerides in the ER. Moreover, FATP1 and DGAT2 complex maintain the ER-LD MCSs and facilitates LD expansion by coupling the synthesis and deposition of triglycerides into LDs. ORP5 plays an important role in transferring PC from the ER to LDs *via* ER-LD MCSs. Mitochondria-LD interactions also play an important role in maintaining lipid homeostasis. Perilipin5 and DGAT2 in LDs recruit the mitochondria to promote the expansion of LDs by providing ATP for triglyceride synthesis. In addition, the interaction of M1 spastin on LDs with ABCD1 on peroxisomes promotes fatty acid transfer from LDs to peroxisomes for β‐oxidation. Dysfunctional growth and expansion of LDs leads to the accumulation of fatty acids, which then react with ROS and produce lipid peroxides, thereby resulting in ER stress, increased mitochondrial ROS, and cellular senescence.

Lipotoxicity is caused by the accumulation of lipids, particularly fatty acids 179. When cellular fatty acids exceed the requirements of synthesis and catabolism, they can be stored in the form of triacylglycerol in LDs to avoid lipotoxicity 179. However, the capacity of triacylglycerol buffering is limited 179, 183. When the limited triacylglycerol buffering capacity is saturated, accumulated lipids react with ROS, especially mitochondrial ROS, thereby producing large quantities of lipid peroxides. Interestingly, continuous production and accumulation of lipid peroxides cause serious lipid toxicity-related damage to mitochondrial structure and function, and induce a rapid increase in ROS levels, thereby contributing to cellular senescence 184. Moreover, continuous production and accumulation of lipid peroxides can induce ER stress 179, 182, which also contributes to cellular senescence. It has been shown that triacylglycerol accumulates in replicative senescent cells. This is a cellular mechanism that prevents lipotoxicity in senescent cells 185. As LDs play primary roles in lipid storage and homeostasis 179, the biogenesis and expansion of LDs, which are regulated by organelle interactions, play significant roles in cellular senescence. It has been shown that LD contact sites play an important role in fatty acid synthesis, storage, release, and breakdown 186. Inhibition of LD biogenesis during rapid fatty acid release leads to fatty acid-mediated mitochondrial depolarization 81, which increases the release of mitochondrial ROS 187, and leads to cellular senescence (Fig.4).

LDs originate from ER 182. Most neutral lipid and phosphatidylcholine (PC) synthesizing enzymes are absent in LDs, while they exist in the ER, implying that the interaction between LDs and the ER is essential for lipid metabolism 35. LDs widely interact with the ER *via* membrane bridges and MCSs. The ER-localized protein ORP5 plays an important role in the transfer of PC from the ER to LDs in ER-LD MCSs 188. The tether proteins of ER-LD MCSs include ER-localized fatty acid transport protein 1 (FATP1) and LD-localized diacylglycerol acyltransferase 2 (DGAT2), the ER-associated NAG-RINT1-ZW10 (NRZ) tethering complex and their associated SNAREs (Syntaxin18, Use1, BNIP1), and LD-localized Rab18 8, 35, 189. Rab18 is associated with lipolysis and lipogenesis, and its overexpression induces a close apposition of the ER and LDs. It has been shown that the Rab18-NRZ-SNARE interaction facilitates LD growth and maturation by regulating the synthesis of triglycerides in the ER 35, 189. The FATP1 and DGAT2 complexes maintain the ER-LD MCSs and facilitate LD expansion by coupling the synthesis and deposition of triglycerides into LDs 35, 190.

Except for ER-LD MCSs, there are membrane bridges where the LD monolayer appears to be directly continuous with the ER bilayer, playing a significant role in maintaining the structure and biogenesis of LDs and facilitating the incorporation of neutral lipids and membrane proteins into LDs 8, 35. Notably, triacylglycerol synthesis enzymes are required for LD growth and expansion, and they can be transferred from the ER into LDs *via* membrane bridges 180. Moreover, the ER protein seipin plays an important role in protein transfer from the ER to LDs, LD biogenesis, and the regulation of the local lipid environment 1, 35, 191.

The interactions between the mitochondria and LDs also play an important role in maintaining lipid homeostasis. In the presence of excess fatty acids in the cell, LD coat proteins perilipin5, and DGAT2, which catalyze the final step in triglyceride synthesis 192, recruit mitochondria to LDs to provide ATP to promote the expansion of LDs, thereby protecting against lipotoxic insult 81, 193.

It has been shown that 10% of LDs form contacts with peroxisomes 73. The peroxisome-LD MCSs may link LD-mediated lipolysis to fatty acid β‐oxidation in peroxisomes. LDs protein M1 spastin interacts with ATP binding cassette subfamily D member 1 (ABCD1), a peroxisome protein, to promote peroxisome-LD MCS formation and facilitate direct channeling of fatty acids across the boundaries of organelles to protect against lipotoxic insult 93, 97.

## 4. A strategy for delaying senescence based on organelle interaction

### 4.1 Maintaining calcium homeostasis

**Figure 5:**
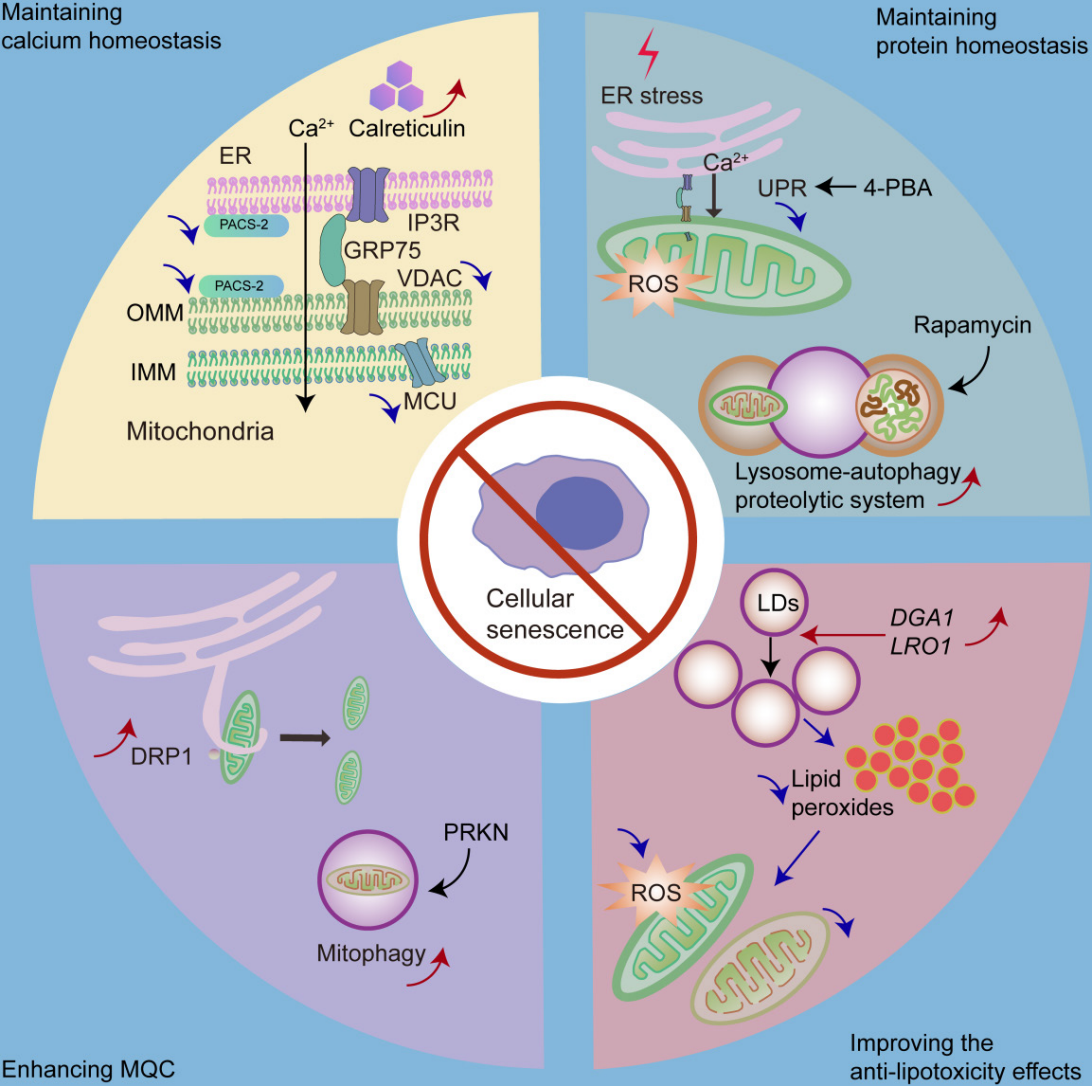
There are some strategies for delaying senescence with respect to organelle interaction, including maintaining calcium homeostasis, maintaining protein homeostasis, enhancing MQC, and improving anti-lipotoxicity effects. We can decrease mitochondrial calcium intake by repressing the expression of IP3R, MCU or PACS-2, and increasing the expression of calreticulin. Moreover, we can inhibit the overactivated UPR by 4-PBA to delay cellular senescence. Promoting lysosome-mediated autophagy by rapamycin is also effective. Furthermore, we can increase mitochondrial fission or mitophagy *via* DRP1 or PRKN, respectively, to enhance MQC. Moreover, by overexpressing DGA1 (encodes DGAT homologue in yeast) and LRO1 (encodes phospholipid:diacylglycerol O-acyltransferase in yeast), we can increase the number of LDs to decrease mitochondrial ROS and mitochondrial fragmentation caused by high amounts of fatty acids.

Mitochondrial calcium homeostasis plays an important role in maintaining mitochondrial function. Abnormal organelle interactions lead to excessive calcium intake into the mitochondria, thereby leading to cellular senescence. Thus, maintaining mitochondrial calcium homeostasis may be a reasonable way to retard cellular senescence. Notably, knockdown of MCU can prevent cellular senescence 115. Furthermore, IP3-induced IP3R activation induces premature senescence 117, whereas IP3R absence decreases the progression of aging 115 (Fig.5).

Mitochondrial calcium homeostasis can be regulated through ER-mitochondria MCSs. The increased number and area of ER-mitochondria MCSs leads to enhanced calcium transfer from the ER to the mitochondria. It has been shown that the downregulation of phosphofurin acidic cluster sorting protein 2 (PACS-2), a key regulator of ER-mitochondria MCSs, retards mitochondrial calcium accumulation 194. While an increase in the ER-mitochondria contact leads to cellular senescence 116, the ablation of ER-mitochondria MCS tethers delays cellular senescence 116.

ER is the major storage compartment for intercellular calcium 107. ER calcium homeostasis is closely related to mitochondrial calcium homeostasis 195. There is an inverse relationship between calcium storage in the ER and the mitochondria 195. Notably, a large amount of calcium is buffered by calcium-binding proteins, such as calsequestrin or calreticulin, in the ER 196. It has been shown that decreased expression of calreticulin is related to aging 197, while overexpression of calreticulin enhances ER calcium storage and inhibits the activity of IP3R, thereby regulating mitochondrial calcium homeostasis 195.

### 4.2 Maintaining protein homeostasis

Overactivation of the UPR is positively associated with senescence 99. One possible mechanism could be that UPR leads to an imbalance in mitochondrial calcium homeostasis. UPR activation is associated with cellular protection; however, overactivation of the UPR is associated with pathological processes. UPR inhibition also shows therapeutic value 195. It has been shown that the inhibition of UPR retards cellular senescence 99. For example, the chemical chaperone 4-phenylbutyric acid (4-PBA), a UPR inhibitor, reduces the number of senescent cells in a premature tubular epithelial cell senescence model 198.

In addition, lysosome-mediated autophagy plays an important role in maintaining cellular protein homeostasis and preventing cellular senescence; however, it is impaired in senescent cells 199. Autophagy activation has been shown to inhibit stress-induced senescence 146, 200. As a result, enhancing the function of lysosome-autophagy proteolytic system may be a strategy to delay senescence. It has been shown that restoring autophagy in stem cells can prevent cellular senescence and maintain stemness 144. Furthermore, rapamycin, an autophagy inducer, can protect against oxidative stress-induced senescence 146.

### 4.3 Enhancing MQC

Mitochondrial dysfunction and MQC failure play significant roles in cellular senescence 166, 201. Thus, enhancing MQC may be a therapeutic strategy against cellular senescence. Of note, DRP1 plays a significant role in mitochondrial fission by generating a constriction ring around the mitochondria 166. Increased p53 levels in senescent cells inhibit DRP1 translocation into the mitochondria 202, while a previous report has shown that the increased expression of DRP1 promotes mitochondrial fission and delays cellular senescence 203, 204. In addition, Parkin RBR E3 ubiquitin protein ligase (PRKN) plays a crucial role in regulating mitophagy 205; hence, overexpression of PRKN could prevent cellular senescence by increasing mitophagy 205.

### 4.4 Improving anti-lipotoxicity effects

Since LDs play important roles against lipotoxicity, increasing the number of LDs may be an appropriate way to ameliorate cellular senescence. The overexpression of DGA1 (encodes DGAT homologue in yeast) and LRO1 (encodes phospholipid:diacylglycerol O-acyltransferase in yeast) 206, both of which are associated with triacylglycerol synthesis, leads to an increased number of LDs and reduced mitochondrial fragmentation as well as mitochondrial ROS production, ultimately contributing to the alleviation of replicative senescence 207.

## 5. Conclusions

The essential reactions in eukaryotic cells are compartmentalized in membrane-bound organelles, which allow the segregation of incompatible biological processes with tailored microenvironments 1, 25. However, the cell is a complicated biological system, and organelles are not isolated entities 86; they need to actively communicate and interact with each other to maintain cell homeostasis 1, 208. For example, peroxisomes convert glyoxylate and alanine into glycine and pyruvate; then, glycine is transported into the mitochondria for oxidation and the detoxification of glyoxylate 91. The exchange of metabolites and information between organelles depends on vesicular trafficking pathways and MCSs 1.

Cellular senescence is a process of permanent cell-cycle arrest. It involves cellular calcium homeostasis, protein homeostasis, and MQC and is regulated by organelle interactions. In this review, we provide important clues to the intervention strategies for cellular senescence from a new perspective, including maintaining calcium homeostasis, maintaining the protein homeostasis, enhancing MQC, and improving anti-lipotoxicity effects.

Dysfunctional organelle interactions not only lead to cellular senescence, but also affect other important processes, such as the imbalance of cellular energy metabolism. Mitochondria are the main energy producers 209. Their activities, especially ATP production, are regulated by calcium signaling 209. In particular, IP3R-mediated calcium transfer in ER-mitochondria MCSs plays an important role in maintaining basal mitochondrial metabolism 209, and the inhibition of IP3R activity leads to the impaired cellular energy metabolism under normal cellular conditions 209. Peroxisomes and mitochondria act together in the degradation of fatty acids 91. Dysfunctional mitochondria can replenish the TCA cycle *via* the RTG pathway, where peroxisomes convert fatty acids into acetyl-CoA 93, 94. Defects in peroxisomes have an impact on mitochondrial pathology due to the impairment of fatty acid metabolism 210. Thus, the organelles cooperatively form an interactive network to maintain cell homeostasis and accomplish a series of biological processes. Further understanding of the relevance of organelle interactions would certainly broaden our horizons for the understanding of mechanical and therapeutic exploration of complicated cell behavior. However, this is not limited to cellular senescence. Hence, our review provides important clues for a deeper understanding of cell function from a new perspective.

## Abbreviation

ABCD1: ATP binding cassette subfamily D member 1
ACBD2: acyl-CoA binding domain protein 2
ACBD5: acyl-CoA binding domain protein 5
AMPK: AMP-activated protein kinase
ATF6α: activating transcription factor 6 alpha
ATG5: autophagy-related gene 5
ATG14: autophagy-related gene 14
BAP31: B‐cell receptor‐associated protein 31
CERT: ceramide-transfer protein
CRAC: Ca^2+^ release-activated Ca^2+^
DDR: DNA damage response
DGAT2: diacylglycerol acyltransferase 2
DRP1: dynamin-related protein-1
ECI2: enoyl-CoA-δ isomerase 2
ER: endoplasmic reticulum
E-Syts: extended synaptotagmin-like proteins
FATP1: fatty acid transport protein 1
FFAT: phenylalanine-phenylalanine acidic tract
FIS1: mitochondrial fission 1
GRP75: glucose‐regulated protein 75
IMM: inner mitochondrial membrane
IP3R: inositol 1,4,5-triphosphate receptor
IRE1α: inositol requiring enzyme 1 alpha
IST1: IST1 factor associated with the endosomal sorting complex required for transport-III
LAMP1: lysosomal associated membrane protein 1
LDL: low-density lipoprotein
LDs: lipid droplets
MCOLN1: mucolipin 1
MCS: membrane contact site
MCU: mitochondrial calcium uniporter
MDV: mitochondrial-derived vesicle
Mfn2: mitofusin 2
MQC: mitochondrial quality control
mtDNA: mitochondrial DNA
NRZ: NAG-RINT1-ZW10
OMM: outer mitochondrial membrane
ORPs: oxysterol-binding protein-related protein family
OSBP: oxysterol-binding protein
ORP1L: oxysterol-binding protein-related protein 1
PACS-2: phosphofurin acidic cluster sorting protein 2
PC: phosphatidylcholine
PDZD8: PDZ domain‐containing protein 8
PE: phosphatidylethanolamine
PERK: RNA-dependent protein kinase-like ER kinase
PGC-1α: proliferator-activated receptor gamma coactivator 1-alpha
PI: phosphatidylinositol
PITPNM1: phosphatidylinositol transfer protein membrane associated 1
PI(4)P: phosphatidylinositol 4-phosphate
PI(4,5)P2: phosphatidylinositol‐4,5‐bisphosphate
PM: plasma membrane
PRKN: Parkin RBR E3 ubiquitin protein ligase
PS: phosphatidylserine
PTPIP51: protein tyrosine phosphatase interacting protein 51
ROS: reactive oxygen species
RTG: retrograde
RyR1: ryanodine receptor 1
SASP: senescence-associated secretory phenotype
SERCA: sarco/ER Ca^2+^-ATPase
STARD3: steroidogenic acute regulatory protein-related lipid transfer domain-3
STIM1: stromal interaction molecule 1
STX17: syntaxin 17
Syt7: Synaptotagmin-7
TBC1D15: TBC domain family member 15
TCA: tricarboxylic acid
TFEB: transcription factor EB
TMBIM6: transmembrane BAX inhibitor motif containing 6
TMEM: transmembrane
TNTs: tunneling nanotubes
TRPML1: transient receptor potential mucolipin 1
UPR: unfolded protein response
VAPB: vesicle-associated membrane protein-associated protein B
VAPs: vesicle-associated membrane protein-associated proteins
VDAC: voltage-dependent anion-selective channel protein
4-PBA: 4-phenylbutyric acid
